# Resequencing and Association Analysis of *CLN8* with Autism Spectrum Disorder in a Japanese Population

**DOI:** 10.1371/journal.pone.0144624

**Published:** 2015-12-14

**Authors:** Emiko Inoue, Yuichiro Watanabe, Jingrui Xing, Itaru Kushima, Jun Egawa, Shujiro Okuda, Satoshi Hoya, Takashi Okada, Yota Uno, Kanako Ishizuka, Atsunori Sugimoto, Hirofumi Igeta, Ayako Nunokawa, Toshiro Sugiyama, Norio Ozaki, Toshiyuki Someya

**Affiliations:** 1 Department of Psychiatry, Niigata University Graduate School of Medical and Dental Sciences, Niigata, Japan; 2 Department of Psychiatry, Nagoya University Graduate School of Medicine, Nagoya, Aichi, Japan; 3 Division of Bioinformatics, Niigata University Graduate School of Medical and Dental Sciences, Niigata, Japan; 4 Oojima Hospital, Sanjo, Niigata, Japan; 5 Department of Child and Adolescent Psychiatry, Hamamatsu University School of Medicine, Hamamatsu, Shizuoka, Japan; Emory University School Of Medicine, UNITED STATES

## Abstract

Rare variations contribute substantially to autism spectrum disorder (ASD) liability. We recently performed whole-exome sequencing in two families with affected siblings and then carried out a follow-up study and identified ceroid-lipofuscinosis neuronal 8 (epilepsy, progressive with mental retardation) (*CLN8*) as a potential genetic risk factor for ASD. To further investigate the role of *CLN8* in the genetic etiology of ASD, we performed resequencing and association analysis of *CLN8* with ASD in a Japanese population. Resequencing the *CLN8* coding region in 256 ASD patients identified five rare missense variations: g.1719291G>A (R24H), rs201670636 (F39L), rs116605307 (R97H), rs143701028 (T108M) and rs138581191 (N152S). These variations were genotyped in 568 patients (including the resequenced 256 patients) and 1017 controls. However, no significant association between these variations and ASD was identified. This study does not support a contribution of rare missense *CLN8* variations to ASD susceptibility in the Japanese population.

## Introduction

Autism spectrum disorder (ASD) is a neurodevelopmental disorder characterized by early-onset difficulties in social communication and unusually restricted, repetitive behavior and interests [1]. Genetic risk of ASD has been suggested to involve the combined effects of many common variations of small effect, as well as rare variations of large effect [2].

Our recent whole-exome sequencing (WES) study in two families with affected siblings (S1 Fig) and a follow-up study identified ceroid-lipofuscinosis, neuronal 8 (epilepsy, progressive with mental retardation) (*CLN8*) as a potential genetic risk factor for ASD [3]. In one family, a rare heterozygous missense variation, R24H, was transmitted from an affected father to three affected sons and thus co-segregated with ASD. In the follow-up study (550 patients and 1017 controls), heterozygous *CLN8* R24H was identified in one patient and one control. R24H had a higher mutant allele frequency in patients (0.09%) compared with controls (0.05%), although the association was not significant.

Certain homozygous or compound heterozygous *CLN8* mutations cause two distinct variants of neuronal ceroid lipofuscinosis-8 (OMIM#600143): Northern epilepsy variant (OMIM#610003), also known as progressive epilepsy with mental retardation (EPMR; [4]), and a more severe form of variant late-infantile neuronal ceroid lipofuscinosis [5]. For example, R24G co-segregated with EPMR; all 22 patients were homozygous, and all 10 parents and 19 of 28 healthy siblings were heterozygous [4]. To our knowledge, no studies investigating neuronal ceroid lipofuscinosis-8 have described heterozygous carriers who manifest psychiatric traits compatible with ASD and/or intellectual disability. However, other rare heterozygous *CLN8* variations may confer increased susceptibility to ASD.

CLN8 is involved in cell proliferation during neuronal differentiation and in protection against neuronal cell apoptosis [6]. Teratocarcinoma P19 cells expressing the deletion mutant *Cln8* K61del showed an increased proliferation rate throughout neuronal differentiation [6]. In P19 cells, *Cln8* mutations (K61del, A30P, Y158C and Q194R) and *Cln8* silencing by small hairpin RNA increased apoptosis rates induced by *N*-methyl d-aspartate [6]. Of note, several lines of evidence suggest that abnormal neural cell proliferation and/or death are implicated in the pathophysiology of ASD [7–9]. A longitudinal and cross-sectional magnetic resonance imaging study of autism cases demonstrated early brain overgrowth during infancy and the toddler years, followed by an accelerated rate of size reduction and perhaps degeneration from adolescence to late middle age [7]. A postmortem study showed higher prefrontal neuron counts and brain weight in children with autism compared with controls [8]. Levels of anti- and pro-apoptotic proteins are reduced and increased, respectively, in the postmortem brain of ASD patients [9].

To further investigate the role of *CLN8* in the genetic etiology of ASD, we resequenced the *CLN8* coding region in 256 ASD patients, and then performed an association study with 568 patients and 1017 controls.

## Materials and Methods

### Ethics Statement

This study was approved by the Ethics Committee on Genetics of Niigata University School of Medicine, and the Ethics Committee of the Nagoya University Graduate School of Medicine and associated institutes and hospitals. This study was conducted in accordance with the Declaration of Helsinki. Written informed consent was obtained from all participants and/or their families.

### Participants

All participants were unrelated and of Japanese descent. The Niigata sample consisted of 256 patients with ASD (202 males and 54 females; mean age, 19.0 [SD 9.2] years) and 667 control individuals (341 males and 326 females; mean age, 38.3 [SD 10.8] years). The sample included 241 patients and 667 controls involved in the study by Egawa *et al*. [3]. Patient and control groups were not sex- or age-matched. Each participant was subjected to psychiatric assessment, as previously described [3]. In brief, patients were diagnosed by experienced child psychiatrists according to Diagnostic and Statistical Manual of Mental Disorders, 4th Edition (DSM-IV) criteria for autistic disorder (*n* = 72), Asperger’s disorder (*n* = 116), or pervasive developmental disorder not otherwise specified (PDD-NOS; *n* = 68). The Autism Diagnostic Observation Schedule has not been translated into Japanese. Although the Autism Diagnostic Interview-Revised has been recently translated into Japanese, no training in the use of this interview technique was available to us in Japan. Thus, we were unable to use such standardized tests. Controls were mainly recruited from hospital staff, who showed good social and occupational skills with no self-reported personal or family history (within first-degree relatives) of psychiatric disorders. However, these control individuals were not assessed using structured psychiatric interviews.

The Nagoya sample consisted of 312 patients with ASD (236 males and 76 females; mean age, 19.6 [SD 10.2] years) and 352 control individuals (109 males and 243 females; mean age, 45.9 [SD 10.8] years). These individuals were identical to those in the report of Egawa *et al*. [3]. Patient and control groups were not sex- or age-matched. Cases were included if they met DSM-IV Text Revision criteria for autistic disorder (*n* = 131), Asperger’s disorder (*n* = 89), or PDD-NOS (*n* = 92). Control subjects were selected from the general population and had no history of mental disorders based on questionnaire responses from the subjects during the sample inclusion step.

### Resequencing the *CLN8* coding region

The *CLN8* coding region (RefSeq accession number, NM_018941.3) was resequenced in 256 ASD patients of the Niigata sample by direct sequencing of polymerase chain reaction products, as previously described [10]. Primer sequences used for amplification are listed in S1 Table. Detailed information on amplification conditions is available upon request.

### Genotyping

Rare non-synonymous variations with mutant allele frequencies < 0.01, identified by resequencing, were genotyped in the Niigata and Nagoya samples using the TaqMan 5′-exonuclease assay (Applied Biosystems, Foster City, CA, USA; S2 Table), as previously described [11].

### Statistical analysis

Deviations from Hardy-Weinberg equilibrium were tested using the χ^2^ test for goodness-of-fit. Allelic associations were tested using Fisher’s exact test. A gene-based analysis was performed using the sequence kernel association test (SKAT; http://www.hsph.harvard.edu/skat/) [12]. Files for the analysis were formatted by PLINK (http://pngu.mgh.harvard.edu/purcell/plink/) [13].

A power calculation was performed using the Genetic Power Calculator (http://pngu.mgh.harvard.edu/~purcell/gpc/). Power was estimated using α = 0.05, and assuming a disease prevalence of 0.01 [1].

## Results

**Fig 1:**
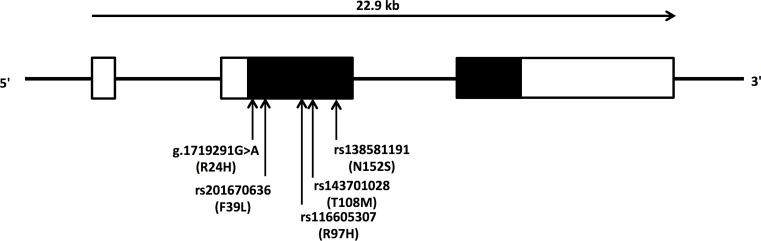
Genomic structure of the ceroid-lipofuscinosis, neuronal 8 (epilepsy, progressive with mental retardation) (*CLN8*) gene. *CLN8* spans approximately 22.9 kb and has three exons (rectangles). Black and white rectangles represent coding and untranslated regions, respectively. A horizontal arrow shows the orientation of transcription. Vertical arrows indicate locations of rare non-synonymous variations identified by resequencing.

Resequencing the *CLN8* coding region in 256 ASD patients of the Niigata sample, we identified five rare non-synonymous variations: g.1719291G>A (R24H), reported in our previous study [3], rs201670636 (g. 1719337T>G; F39L), rs116605307 (g. 1719510G>A; R97H), rs143701028 (g.1719543C>T; T108M) and rs138581191 (g. 1719675A>G; N152S; Table 1; Fig 1).

**Table 1:** Rare non-synonymous *CLN8* variations identified by resequencing.

dbSNP ID	Position^a^	Allele^b^	Exon	Protein	GERP	Function prediction		CADD	MAF	
						PolyPhen-2	SIFT		HGVD	1K (JPT)
-	1719291	G/A	2	R24H	5.09	Probably damaging	Tolerated	25.7	-	-
rs201670636	1719337	T/G	2	F39L	-9.35	Probably damaging	Tolerated	9.4	0.0020	0.0060
rs116605307	1719510	G/A	2	R97H	-7.78	Benign	Tolerated	0.7	0.0013	0
rs143701028	1719543	C/T	2	T108M	5.06	Possibly damaging	Tolerated	22.6	-	0.0060
rs138581191	1719675	A/G	2	N152S	3.9	Possibly damaging	Tolerated	2.8	0.0023	-

CADD, Combined Annotation Dependent Depletion; GERP, Genomic Evolutionary Rate Profiling; 1K, 1000 Genomes; HGVD, the Human Genetic Variation Database; JPT, Japanese in Tokyo; MAF, mutant allele frequency.

^a^Position on chromosome 8 according to GRCh37.

^b^Reference/mutant allele.

These five rare missense *CLN8* variations were genotyped in the Niigata sample (Table 2; S3 Table). In all 256 patients, genotypes of these variations, determined using the TaqMan method, were identical to those obtained by direct sequencing. Genotype distributions of the variations did not deviate significantly from Hardy–Weinberg equilibrium in ASD or control groups (data not shown). All five variations had a higher mutant allele frequency in patients compared with controls (i.e., odds ratios [OR] > 1), although these associations were not significant. We then genotyped the five rare missense *CLN8* variations in the Nagoya sample. Genotype distributions of the variations did not deviate significantly from Hardy–Weinberg equilibrium in ASD or control groups (data not shown). Two variations (F39L and R97H) also had a higher mutant allele frequency in patients compared with controls, although these associations were not significant. When we combined the Niigata and Nagoya samples, all five rare missense *CLN8* variations had a higher mutant allele frequency in patients compared with controls, but were not significantly associated with ASD. A gene-based analysis revealed that there was no significant association between a set of these rare variations and ASD (SKAT *p* = 0.53).

**Table 2:** Association analysis of five rare missense *CLN8* variations with ASD.

Variation	ASD				Control				Allelic *p*	OR	95% CI
	1/1^a^	1/2^a^	2/2^a^	MAF	1/1^a^	1/2^a^	2/2^a^	MAF			
R24H	G/G	G/A	A/A	A	G/G	G/A	A/A	A			
Niigata	255	1	0	0.0020	666	1	0	0.0007	0.48	2.6	0.2–41.8
Nagoya	309	0	0	0	350	0	0	0	-	-	-
Combined	564	1	0	0.0009	1016	1	0	0.0005	1.00	1.8	0.1–28.8
F39L	T/T	T/G	G/G	G	T/T	T/G	G/G	G			
Niigata	255	1	0	0.0020	664	2	0	0.0015	1.00	1.3	0.1–14.4
Nagoya	308	1	0	0.0016	352	0	0	0	0.47	∞	-
Combined	563	2	0	0.0018	1016	2	0	0.0010	0.62	1.8	0.3–12.8
R97H	G/G	G/A	A/A	A	G/G	G/A	A/A	A			
Niigata	255	1	0	0.0020	664	0	0	0	0.28	∞	-
Nagoya	310	1	0	0.0016	351	0	0	0	0.47	∞	-
Combined	565	2	0	0.0018	1015	0	0	0	0.13	∞	-
T108M	C/C	C/T	T/T	T	C/C	C/T	T/T	T			
Niigata	255	1	0	0.0020	665	0	0	0	0.28	∞	-
Nagoya	309	0	0	0	350	1	0	0.0014	1.00	0	-
Combined	564	1	0	0.0009	1015	1	0	0.0005	1.00	1.8	0.1–28.8
N152S	A/A	A/G	G/G	G	A/A	A/G	G/G	G			
Niigata	255	1	0	0.0020	659	1	0	0.0008	0.48	2.6	0.2–41.3
Nagoya	310	0	0	0	352	0	0	0	-	-	-
Combined	565	1	0	0.0009	1011	1	0	0.0005	1.00	1.8	0.1–28.6

ASD, autism spectrum disorder; CI, confidence interval; MAF, mutant allele frequency; OR, odds ratio.

^a^Genotypes: reference and mutant alleles are denoted by 1 and 2, respectively.

Clinical phenotypes of patients with rare missense *CLN*8 variations are shown in Table 3. R24H was identified in a male patient with autistic disorder, selective mutism, and epilepsy (patient #1). This patient corresponds to patient #1 of the Niigata cohort in our previous study [3]. F39L was identified in a male patient with PDD-NOS (patient #2) and in a female patient with autistic disorder, dissociative fugue, and mild mental retardation (patient #3). R97H was identified in two male patients with autistic disorder (patient #4 and patient #5). T108M was identified in a female patient with Asperger’s disorder (patient #6). N152S was identified in a male patient with autistic disorder and moderate mental retardation (patient #7).

**Table 3:** Clinical phenotypes of the ASD patients with rare missense *CLN8* variations.

Clinical phenotype	Patient						
	#1	#2	#3	#4	#5	#6	#7
Variation	R24H	F39L	F39L	R97H	R97H	T108M	N152S
Sex	Male	Male	Female	Male	Male	Female	Male
Age	9	24	21	26	8	10	20
DSM-IV Diagnosis	Autism	PDD-NOS	Autism	Autism	Autism	Asperger’s	Autism
Comorbidity	Selective mutism	-	Dissociative fugue and mental retardation	-	-	-	Mental retardation
Full-scale IQ	No data	No data	57	No data	79	79	46
Epilepsy	+	-	-	-	-	-	-

ASD, autism spectrum disorder; IQ, intelligence quotient; PDD-NOS, pervasive developmental disorder not otherwise specified.

## Discussion

In our previous WES study, *CLN8* R24H was transmitted from an affected father to three affected sons and thus co-segregated with ASD in one family [3]. In the case-control study, R24H was not significantly associated with ASD, although the H allele frequency was higher in patients than in controls [3]. In this study, we resequenced the *CLN8* coding region in 256 ASD patients, and detected five rare missense variations (R24H, F39L, R97H, T108M and N152S). In the combined sample comprising the Niigata and Nagoya samples (568 patients and 1017 controls), which mostly overlapped with the samples in our previous study [3], all five variations had a higher mutant allele frequency in patients compared with controls. In particular, R97H was identified exclusively in two male patients with autistic disorder. However, there was no significant association between rare missense *CLN8* variations and ASD. The results of our previous and present studies do not provide convergent evidence for a contribution of rare missense *CLN8* variations to ASD susceptibility.

*CLN8* encodes a protein that has five transmembrane domains (residues 21–41, 62–84, 103–123, 131–151 and 226–246), a TRAM-LAG1-CLN8 (TCL) domain (residues 62–262), and a C-terminal endoplasmic reticulum (ER)-retrieval signal (residues 283–286) [14]. Genomic Evolutionary Rate Profiling (GERP; http://mendel.stanford.edu/SidowLab/downloads/gerp/) scores for R24H and T108M were 5.09 and 5.06, respectively, indicating that these variations are evolutionarily conserved (Table 1). The functional effects of R24H and F39L were predicted to be ‘probably damaging’ by PolyPhen-2 (http://genetics.bwh.harvard.edu/pph2/), whereas all five rare missense variations were predicted to be ‘tolerated’ by SIFT (http://sift.bii.a-star.edu.sg/index.html). Combined Annotation Dependent Depletion (CADD; http://cadd.gs.washington.edu/home) scores for R24H and T108M were 25.7 and 22.6, respectively, indicating that these variations are predicted to be among the 1% most deleterious. Nevertheless, functional analyses will be required to assess the accuracy of such predictions.

The neurobiology of CLN8 and the pathophysiology of ceroid lipofuscinosis-8 remain poorly understood. CLN8 is an ER resident protein that recycles between the ER and the ER-Golgi intermediate compartment using the C-terminal ER-retrieval signal [15]. The mRNA levels of ER and oxidative stress marker genes are elevated in cultured fibroblasts from patients with neuronal ceroid lipofuscinoses, including Northern epilepsy [16]. These fibroblasts show significant sensitivity to brefeldin-A-induced apoptosis [16]. Levels of ER stress-associated proteins are increased in the brain of the motor neuron degeneration mouse model of ceroid lipofuscinosis-8 at the presymptomatic state [17]. Taken together, these findings suggest that CLN8 dysfunction results in the impairment of ER stress responses and is, therefore, likely to be involved in the pathophysiology of ceroid lipofuscinosis-8.

Our ASD patients with rare missense *CLN8* variations are all heterozygous, while ceroid lipofuscinosis-8 is caused by autosomal recessive mutations. Several other heterozygous *CLN8* missense variations have been identified by WES in 1208 Japanese individuals. These have been registered at http://www.genome.med.kyoto-u.ac.jp/SnpDB/. However, the variations in these Japanese individuals are not registered in the neuronal ceroid lipofuscinosis (NCL) Mutation and Patient Database (http://www.ucl.ac.uk/ncl/mutation.shtml).

Chien *et al*. [18] reported an ASD patient with a 2.4 Mb terminal deletion at 8p23.2-pter encompassing several genes, including *CLN8* and discs, large (Drosophila) homolog-associated protein 2 (*DLGAP2*). Haploinsufficiency of these genes may be implicated in vulnerability to ASD. Subsequently, they performed *DLGAP2* exon resequencing in 515 ASD patients and 596 controls [19]. Among 16 rare missense and nine common variations detected, two common variations were associated with ASD. However, they did not identify loss-of-function (LoF; nonsense, splice site and frameshift) mutations. That was also the case for our *CLN8* resequencing.

Two large-scale WES studies identified promising risk genes for ASD that are enriched for fragile X mental retardation protein targets [20, 21]. The Autism Sequencing Consortium reported that transmission and *de novo* association analysis of LoF and damaging missense variations reveals 22 genes with a false discovery rate < 0.05 in 3871 patients and 9937 controls [20]. The other WES study detected 27 genes with recurrent *de novo* LoF mutations in 2508 families from the Simons Simplex Collection [21]. Among these 22 and 27 genes, 10 overlapped: *ADNP*, *ANK2*, *ARID1B*, *CHD8*, *DYRK1A*, *GRIN2B*, *KATNAL2*, *POGZ*, *SCN2A*, and *TBR1*. WES studies of multiplex families may be fruitful for the identification of rare inherited variations with large effects on ASD risk [22, 23]. Our WES study of two families with affected siblings indicated that *CLN8* R24H co-segregated with ASD in one family, although *CLN8* was not included among promising risk genes for ASD in two large-scale WES studies [20, 21]. In the present study, we performed resequencing and association analysis of *CLN8* in case-control samples. However, we failed to find significant associations between rare missense *CLN8* variations and ASD.

We recognize the limitations of this study. First, our sample size (568 patients and 1017 controls) may not provide adequate statistical power to detect an association between rare missense *CLN8* variations and ASD because the risk allele frequencies were extremely low (0–0.0010 in controls). Assuming a risk allele frequency of 0.001 and a genotypic relative risk for heterozygous risk allele carriers of 5.0 under the dominant model of inheritance, approximately 6000 patients and 6000 controls are needed to adequately detect association with a power of 0.80. Second, the patient groups were younger and had a higher percentage of males than the control groups in the Niigata and Nagoya samples. ASD affects more male than female individuals [1]. Analyzing males and females separately, we found no significant associations between rare missense *CLN8* variations and ASD (data not shown). Third, we were not able to evaluate participants using standardized structured interviews. Accordingly, we could not exclude the possibility that our negative results may be due to misdiagnosis.

## Conclusion

Our present study does not support the contribution of rare missense *CLN8* variations to ASD susceptibility in the Japanese population.

## Supporting Information

S1 Fig Pedigrees of two families, each with three autism spectrum disorder siblings. (A) Family #1. All three siblings (II-1, II-2, and II-3) were diagnosed with Asperger’s disorder. (B) Family #2. There were four affected individuals: a proband (II-1) with Asperger’s disorder, his brother (II-2) with Asperger’s disorder, his brother (II-3) with Asperger’s disorder and borderline intellectual functioning, and their father (I-1) with pervasive developmental disorder not otherwise specified. In family #2, a rare heterozygous missense variation, *CLN8* R24H, was transmitted from the affected father to the three affected sons and thus co-segregated with ASD. Shaded and unshaded symbols indicate affected and unaffected individuals, respectively. Squares and circles represent males and females, respectively.
(TIF)

S1 Table Primer sequences used for resequencing the *CLN8* coding region. (DOC)

S2 Table Probes used for TaqMan SNP assays. (DOC)

S3 Table Genotypes of five rare missense *CLN8* variations for each participant. (XLSX)
