# Supplementary figures and images for: Resequencing and Association Analysis of CLN8 with Autism Spectrum Disorder in a Japanese Population
Source: PLoS One. 2015 Dec 14;10(12):e0144624. doi: 10.1371/journal.pone.0144624 (PMC4682829; doi:10.1371/journal.pone.0144624)

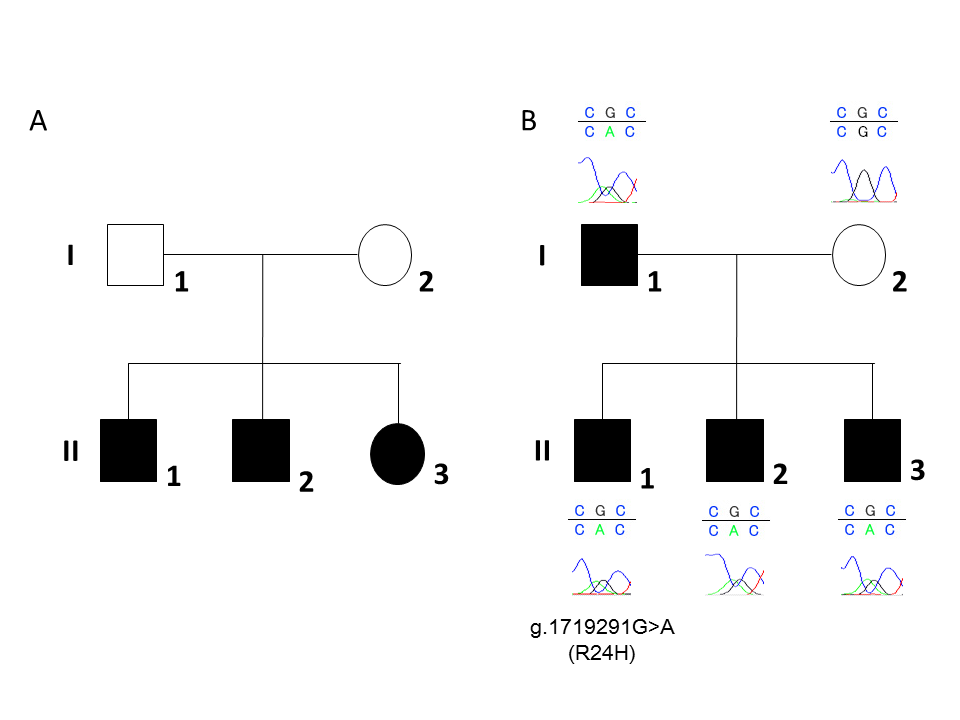

Supplement: S1 Fig — (A) Family #1. All three siblings (II-1, II-2, and II-3) were diagnosed with Asperger’s disorder. (B) Family #2. There were four affected individuals: a proband (II-1) with Asperger’s disorder, his brother (II-2) with Asperger’s disorder, his brother (II-3) with Asperger’s disorder and borderline intellectual functioning, and their father (I-1) with pervasive developmental disorder not otherwise specified. In family #2, a rare heterozygous missense variation, CLN8 R24H, was transmitted from the affected father to the three affected sons and thus co-segregated with ASD. Shaded and unshaded symbols indicate affected and unaffected individuals, respectively. Squares and circles represent males and females, respectively. (TIF) [file pone.0144624.s001.tif]
