# Supplementary material for: Resequencing and Association Analysis of CLN8 with Autism Spectrum Disorder in a Japanese Population
Source: PLoS One. 2015 Dec 14;10(12):e0144624. doi: 10.1371/journal.pone.0144624 (PMC4682829; doi:10.1371/journal.pone.0144624)
Supplement: S1 Table — (DOC) [file pone.0144624.s002.doc]

**S1 Table . Primer sequences used for resequencing the *CLN8* coding region**

| Primer # | Exon | Forward | Reverse |
| --- | --- | --- | --- |
| 1 | 2 | 5′-AGACAAGACACAGTGTAGGG-3′ | 5′-CAGGACATTGTAGCCAGTGA-3′ |
| 2 | 3 | 5′-AAACAGCATGAGCGGGCAAG-3′ | 5′-ATTCAAAAGCCATCATTCGCGG-3′ |
