# Supplementary material for: Resequencing and Association Analysis of CLN8 with Autism Spectrum Disorder in a Japanese Population
Source: PLoS One. 2015 Dec 14;10(12):e0144624. doi: 10.1371/journal.pone.0144624 (PMC4682829; doi:10.1371/journal.pone.0144624)
Supplement: S2 Table — (DOC) [file pone.0144624.s003.doc]

**S2 Table. Probes used for TaqMan SNP assays**

| Variation | Forward primer | Reverse primer | Reporter 1 | Reporter 2 |
| --- | --- | --- | --- | --- |
| R24H | 5'-ATTTTTGACCTGGACTATGCATCCT-3' | 5'-CAAAGCCAGCGACCATCAG-3' | 5'-VIC-CGTGGAGCGGATCC-NFQ-3' | 5'-FAM-CGTGGAGTGGATCC-NFQ-3' |
| F39L | 5'-TCGCTGGCTTTGTCTTCTACTTG-3' | 5'-CCTTCTCTCTGGCCACCAAAG-3' | 5'-VIC-CAGACCACAAAGACGC-NFQ-3' | 5'-FAM-AGACCACCAAGACGC-NFQ-3' |
| R97H | 5'-GGGACCCTGTGCTGCAT-3' | 5'-ATGTGAAACCAGCACCAGTTCT-3' | 5'-VIC-AGGCGCGTGGCCAG-NFQ-3' | 5'-FAM-AGGCGCATGGCCAG-NFQ-3' |
| T108M | 5'-GCAGAACTGGTGCTGGTTTC-3' | 5'-GTGGACTGCAACATTTTCAAAGC-3' | 5'-VIC-CGTTGCTGTCGTGATGT-NFQ-3' | 5'-FAM-CGTTGCTGTCATGATGT-NFQ-3' |
| N152S | 5'-CTCTTTGCCTTTCTTGGGTTTCTTG-3' | 5'-GCAACGTGGTCATAGCTAGATAGTG-3' | 5'-VIC-CAGCTTGGAGATTGACCA-NFQ-3' | 5'-FAM-AGCTTGGAGACTGACCA-NFQ-3' |
